# Changes in LEND trainees’ understanding and application of diversity, equity, inclusion, and justice principles

**DOI:** 10.3389/fped.2024.1446852

**Published:** 2024-11-27

**Authors:** Allison P. Fisher, Lisa M. Gies, Stephanie Weber, Tanya Froehlich, Simon Abimosleh, Neeraja Ravindran, Jennifer Smith

**Affiliations:** ^1^ Division of Developmental Pediatrics, Cincinnati Children's Hospital Medical Center, Cincinnati, OH, United States; ^2^ Division of Developmental and Behavioral Pediatrics, University of Cincinnati College of Medicine, Cincinnati, OH, United States

**Keywords:** curriculum, leadership, developmental disabilities, inclusion, equity, intersectionality, diversity

## Abstract

**Objectives:**

To assess changes in trainees’ knowledge and application of Diversity, Equity, Inclusion, and Justice (DEIJ) concepts after participating in a midwestern academic medical center Leadership Education in Neurodevelopmental and Related Disabilities (LEND) program. LEND is a federally funded year-long program training individuals of various disciplines (e.g., speech pathology, family advocacy, psychology) to better support the health of individuals with disabilities.

**Methods:**

Trainees (*n* = 46) answered questions about their knowledge and application of DEIJ topics before and after program participation in 2021–2022 and 2022–2023. Changes in trainees’ responses were examined using paired-samples *t*-tests.

**Results:**

Thirty-six (78%) participants identified as White, 7 (15%) as Black, 2 (4%) as Asian, and 2 (4%) as more than one race. Three (7%) participants identified as Hispanic/Latino. Over the one-year program, trainees’ perceived knowledge increased [*t*(45) = 5.84, *p* < .001, M_diff_ = .59, *Cohen's D* = 0.86]. Regarding articulating definitions of DEIJ terms, trainees’ summed scores following program participation also improved [*t*(45) = 4.71, *p* < .001, *M_diff_* = 2.37, *Cohen's D* = 0.70]. However, their comfort with addressing prejudicial statements and discussing and combating “-isms” (application of DEIJ skills) did not increase [*t*(45) = 1.74, *p* = .09, *M_diff_* = 0.17, Cohen's D = 0.26].

**Conclusions for practice:**

LEND program participation positively impacted trainees’ perceived DEIJ knowledge and ability to define DEIJ terms. However, future refinements to the curriculum will be needed to improve trainees’ application of skills and to develop a more nuanced understanding of equity, intersectionality, inclusion, and belonging.

## Introduction

Large racial and ethnic disparities exist in the healthcare system, such that racially and ethnically minoritized individuals have less access to high quality healthcare (1–3), experience discrimination (4), do not receive culturally responsive care, and consequently, have poorer health outcomes (3, 5). Other minoritized populations, such as individuals with disabilities (6), individuals identifying as LGBTQIA +  (7), those without English language proficiency (8), and individuals born outside of the United States (9), also experience healthcare disparities. Though large-scale systemic changes are needed to decrease disparities, training healthcare providers to provide culturally responsive care can increase their cultural competence, improve patient satisfaction (10) and promote patients’ health self-efficacy (11) among minoritized populations. Culturally responsive care is defined as care that seeks to understand and address a family's background, their belief systems, and the social contributors of health impacting the family, including institutionalized and personally mediated racism (12).

A recent review identified 89 articles examining the efficacy of training to improve cultural competency and reduce health disparities (13). Educational programs most frequently used simulations (e.g., role plays), discussion groups, lectures, immersion experiences, case-based learning, and reflections, and typically combined educational strategies. Using mixed teaching strategies was associated with greater increases in learners’ knowledge and attitudes; however, most studies used self-reported assessment which may be positively biased (13, 14). In addition, few studies demonstrated improvements in participants’ skills. While many studies increased learners’ knowledge of racially and ethnically minoritized populations and the LGBTQIA + community, as well as encouraged reflection on their own culture and background, few of these studies specifically addressed disability.

Culturally responsive care is particularly important for individuals with disabilities, who typically interact with the healthcare system more frequently than individuals without disabilities (15, 16). Unfortunately, disability remains underrepresented in healthcare education (17, 18), leaving many healthcare providers unprepared to work with this population (19). Consequently, individuals with disabilities frequently face ableism in the healthcare system, such as a lack of physical and cognitive accessibility and discrimination (20–24). Furthermore, despite a well-documented history of unequal healthcare access for individuals with disabilities, the National Institute of Minority Health and Disparities only officially recognized people with disabilities as a population with health disparities in September 2023. The exclusion of and discrimination towards individuals with disabilities contributes to the lack of knowledge and interventions aimed at reducing disparities this population faces, particularly for individuals who also belong to other marginalized groups (e.g., racial, ethnic, sexual, and gender minorities).

Kimberlé Crenshaw introduced the concept of holding multiple marginalized identities through her coining of the term “intersectionality.” Intersectionality refers to “the precise nature of discrimination that occurs when multiple axes of identity are vulnerable to oppression” (25). This concept is crucial for understanding and addressing healthcare inequities faced by individuals with disabilities, as (1) individuals with disabilities often hold identities beyond their disability (or disabilities) that shape their lived experiences, and (2) individuals with minoritized identities are more likely to have disabilities, often as a result of the discrimination and exclusion they encounter (26–28). Therefore, training aimed at promoting culturally responsive care for providers serving individuals with disabilities would benefit from incorporating intersectionality-based concepts to address the health inequities this population faces (29).

Leadership Education in Neurodevelopmental and Related Disabilities (LEND) programs are uniquely poised to train healthcare professionals in culturally responsive care for individuals with disabilities, given their focus on equity and interdisciplinary collaboration. Funded by the Maternal Child Health Bureau (MCHB), these interdisciplinary programs aim to enhance the health and well-being of individuals with disabilities across the lifespan, from infancy to adulthood. With 60 programs across the United States, LEND recruits and trains graduate-level students in fields such as medicine, psychology, and occupational therapy, as well as individuals with developmental disabilities and their family members.

LEND training is guided by the Maternal and Child Health (MCH) Leadership Competencies (30). A key competency is Diversity, Equity, Inclusion, and Accessibility, which aims to ensure that all people and communities are treated with respect, reduce barriers to equity, decrease disparities, and improve health outcomes (30). The interdisciplinary approach of LEND also ensures that the voices of individuals with disabilities and their allies are represented throughout the training process.

Studies have shown that LEND training enhances trainees’ self-efficacy and leadership competencies, as observed by both faculty and trainees (31–33). Research also suggests that healthcare providers trained through LEND are more likely to work with underserved populations than those who have not participated in the program (34). However, to date, no studies have specifically examined LEND's impact on Diversity, Equity, Inclusion, and Justice (DEIJ) competencies. The current study seeks to fill this gap by evaluating how LEND curricula influence trainees’ knowledge and application of DEIJ principles.

## Method

This study was deemed exempt from human subjects research by Cincinnati Children's Hospital Medical Center's Institutional Review Board.

### The Cincinnati Children's LEND training program

The Cincinnati Children's LEND program is a one-year program in which learners participate in over 300 h of training. Training is centered around adult learning, including didactic lectures, evidence-based case presentations, interactive/simulated learning experiences, interdisciplinary clinical care, leadership skills development, and cultural humility activities. In addition, trainees participate in eight or more hours of discipline-specific experiences per week.

The Cincinnati Children's LEND program has also continuously revised its DEIJ curriculum in response to learning from materials created by people with disabilities and other marginalized identities and updated research about the impact of systemic inequity on people with disabilities. The DEIJ curriculum was developed using the MCH conceptual framework. The MCH Leadership Competencies are organized within a conceptual framework in a “progression from self to wider community demonstrating the widening contacts, broadening interests, and growing influence that MCH leaders can experience over their career” (30). The conceptual framework develops and progresses from “self,” focusing on increasing knowledge and reflection, to “others,” in which leadership extends to coworkers, colleagues, students, and patients, to the “wider community,” broadening the impact of leadership to organizations and systems. The Cincinnati Children's LEND DEIJ curriculum centers around these leadership competencies, with particular emphasis on the MCH *Diversity, Equity, Inclusion and Accessibility* competency, *cultural humility* (35) and *structural competency*, which is the recognition of the economic, political, and social factors that produce health disparities and contribute to disease (36).

In line with the MCH Leadership Competencies and conceptual framework, the following DEIJ-related training goals were developed: “At the end of the training year, trainees should be able to (1) define equity in healthcare, (2) describe historical roots that drive inequity today as well as current policies and practices that maintain inequity, (3) identify the impact of their intersecting identities and privilege on lived experiences and interactions with others, (4) examine their own implicit bias, and (5) identify ways to address systemic inequality in healthcare.”

Throughout the year, trainees participated in five, 2-h DEIJ sessions. Each DEIJ session was led by professionals with extensive knowledge and teaching experience in the field of DEIJ, many from marginalized populations themselves and/or who were also trained in the LEND program. Some session leaders are LEND faculty while others are outside consultants. In the first session, which focuses on knowledge building and self-reflection, trainees reflected on their intersecting identities, considering how their identities afford or do not afford them privilege. To build a foundational knowledge base, the second session focused on developing greater awareness of inequity in healthcare and ways to increase equitable care (e.g., person-centered care). The third session continued to emphasize self-reflection, while broadening the sphere to “others,” describing implicit and explicit bias and providing trainees with microintervention strategies to respond to microaggressions. The fourth and fifth sessions addressed the “wider community” by asking students to reflect on community, systems-level, and policy changes needed to increase access to and quality of health care. DEIJ content was also infused into the curriculum throughout the year (see Table 1 for examples).

**Table 1:** Examples of DEIJ throughout LEND curriculum.

Training domain	Lecture title	Objectives	Activities
Leadership: Individual and team-based leadership skill development	Values in Disabilities	Recognize history of disability rights and ways attitudes towards disability have changed over time	Small and large group reflection—students reflected on disability representation in the media
Core curriculum: Broad overview of disability topics	Intellectual and developmental disabilities	Describe different conceptualizations of intellectual and developmental disabilities	Small and large group reflection—Students reflected on a TEDTalk by a Black woman with an intellectual disability, who described her experiences of marginalization and resilience
Seminar in Evidence Based Methods: Team-based research projects	Quantitative methods	Identify the research cycle, ways biases impact research, and the importance of partnering with communities to engage in research	Article review—Students critically reviewed a peer-reviewed journal article to identify the impact of racism on research questions, methods, data analysis, and interpretation
Evidence-Based Case Discussions: Series of seven case series scenarios	ADHD, Down Syndrome, Fragile X, Developmental Coordination Disorder, Cortical Visual Impairment, Spina Bifida	Understand the prevalence and impact of different diagnoses on individuals and families; reflect on the lived experiences of individuals with different conditions	Journaling exercise—Students critically examined peer-reviewed journal article for each lecture and provided written responses describing the impact of neighborhood, community factors/resources, and social factors (e.g., discrimination) on individuals with this type of condition or disability

We used evidence-based teaching methods to promote growth in the four major tenets of cultural humility (see Table 2). Aligned with previous successful curricula, we paired lectures with active learning strategies, such as group discussions and reflection activities (13). For example, in a lecture discussing how to create accessible presentations, trainees were presented with case scenarios of groups for which they would design an accessible presentation (e.g., a group of deaf students, a group of racially diverse family advocates). Students first reflected on ways to adapt the presentation and subsequently discussed ideas for adaptation in small groups.

**Table 2:** Four tenets of cultural humility addressed in the LEND curriculum.

Tenet	Definition
Awareness^a^	Awareness or insight into trainees’ privileged identities, marginalized identities, biases, and communication with culturally diverse individuals
Attitudes	Understanding personal values and belief systems and the way in which upbringing and lived experiences shape them
Knowledge	Awareness of the impact of systemic inequity on marginalized families and knowledge of one's own behaviors when interacting with those from a different culture
Skills	Integrating cultural humility and advocacy for equity in daily practice (e.g., asking families about structural barriers)

^a^ Definitions adapted from Tervalon, Murray-Garcia to reflect learning objectives of LEND.

In addition, we used case-based learning, including de-identified case vignettes and personal case presentations from families of children with disabilities to increase trainees’ knowledge and awareness (37–39). Videos were used to amplify the voices of marginalized individuals, with videos demonstrating individuals’ perspectives and lived experiences. We also played videos of experts discussing research and providing information on topics such as equity, environmental racism, disability justice, and the school-to-prison pipeline. Videos, in combination with other educational strategies, have been associated with greater knowledge (40–43), awareness (40, 43, 44), and skills (13). Trainees also periodically prepared for sessions with readings or read short articles during sessions, including research articles related to case-based discussions and articles sharing individuals’ experiences (45). Throughout the year, sessions incorporated educational technology, such as polls and live quizzes, to increase reflection and engagement (46).

### Measures

Trainees were asked to complete the following measures before and after participation in the LEND program. All survey responses were collected through REDCap and were anonymous. To seek iterative feedback for program improvement, pre-post assessment surveys are a standard component of LEND. Therefore, participants were not compensated for completing the measures for this study. Trainees first completed a demographic survey to obtain information about their race, ethnicity, and other sample characteristics. Due to the lack of validated measures in this area, the following measures were created for this study.

#### DEIJ perceived knowledge and application

We asked participants to indicate to what extent they agreed with nine statements related to understanding and combating structural inequity (e.g., racism, sexism), on a 6-point Likert scale from 1=“strongly agree” to 6=“strongly disagree.” Similar to the DEIJ terms, authors created these questions because they are direct targets of the LEND DEIJ training goals and are addressed in the curriculum. The questions were developed by LEND faculty and staff (AF, SW, JS) and reviewed by the department's anti-racism training program, who are trained in DEIJ education and implement DEIJ training curriculum across Cincinnati Children's Division of Developmental and Behavioral Pediatrics (TF, NR, SA). Six of the nine statements asked trainees about their perceived understanding of biases, equity, culture, privilege, intersectionality, and ableism and were considered Perceived Knowledge prompts. Three of the nine statements assessed trainees’ perceived comfort with confronting and discussing prejudice and discrimination and were considered Application prompts. Separate mean Perceived Knowledge and Application domain scores were created by summing the scores for each item in a domain and then dividing by that domain's number of items. A mean Perceived Knowledge and Application total score was created by summing the scores for all nine prompts and dividing by nine.

### DEIJ knowledge

Trainees were asked to define nine DEIJ terms: equity, privilege, intersectionality, ableism, microaggressions, systems-centered language, diversity, inclusion, and belonging. They were given the choice to select the following response options, “I am unsure what this word means,” and “I have not heard of this word.” The authors chose these terms because of their importance and relevance to culturally responsive leadership and because they are targets of the LEND DEIJ training goals. Therefore, these concepts were addressed throughout the LEND curriculum. For example, we discussed “belonging,” creating a safe space where individuals with disabilities can be their authentic selves, in several LEND training sessions.

The Knowledge of DEIJ terms measure was scored by comparing trainee definitions of DEIJ terms to standard definitions for each item (47). Responses were marked as “correct” (2 points), “partially correct” (1 point), and “incorrect” (0 points), similar to scoring on the Weschler Intelligence Scale for subtests assessing individuals’ vocabulary and verbal reasoning (48). Responses were scored as “partially correct” if they included one or more aspects of the correct definition but did not provide a complete and thorough definition. For example, if a trainee stated that “privilege” was an advantage but did not include that privilege is not earned, the definition was marked as “partially correct.”

### Participants

Trainees answered questions before and after completion of the LEND program in the 2021–2022 (*n* = 24) and 2022–2023 (*n* = 22) training year.

### Data analysis

Because there were no differences between trainees’ responses in 2021–2022 and 2022–2023 on the outcome measures, we combined responses across the two LEND training years. We used paired-samples *t*-tests to examine pre-post changes in the Perceived Knowledge and Application total score (average of all nine questions) and average “Perceived Knowledge” and “Application” domain scores.

For the knowledge of DEIJ terms, two independent coders from the research team evaluated and scored trainees’ responses to each term. They met and came to consensus on discrepant ratings without disagreement. A third coder reviewed 25% of trainees’ responses for inter-rater reliability in year 1 and 10% in year 2, which was substantial (Cohen's Kappa = .73). Trainees’ scores on each of the nine terms were summed to create a Knowledge total score.

To assess changes in Knowledge of DEIJ terms, we summed trainees’ response scores for each term at the pre- and post survey administration and conducted a paired-samples *t*-test to examine changes in trainees’ total scores across the nine terms. We described trainees’ “post-LEND” definition responses to better identify trainees’ understanding after participating in the LEND training program and where changes to the curriculum may be warranted to improve trainees’ knowledge of DEIJ terms.

## Results

Most participants were White (78.3%), female (91.3%), and came from a variety of disciplines (see Table 3). Five (11%) trainees identified as having a disability, one (2.2%) as having a special healthcare need, eight (17.5%) as a parent of a child with a disability or special healthcare need and 15 (33%) as a non-parent family member of a person with a disability or special health care need (e.g., sibling of a person with a disability).

**Table 3:** Demographic characteristics of the sample.

Characteristic, N (%)	2022, *n* = 24	2023, *n* = 22	Total sample
Gender			
Female	22 (92%)	20 (91%)	42 (91.3%)
Male	1 (4%)	2 (9%)	3 (6.5%)
Non-binary	1 (4%)	0 (0%)	1 (2.2%)
Race			
White	19 (79%)	17 (74%)	36 (78.3%)
Black/African American	3 (8%)	4 (17%)	7 (15.2%)
Asian	1 (4%)	1 (4%)	2 (4.3%)
More than one race	1 (4%)	1 (4%)	2 (4.3%)
Ethnicity			
Hispanic/Latino	2 (8%)	1 (4%)	3 (6.5%)
Non-Hispanic	22 (92%)	21 (96%)	43 (93.5%)
Education			
Certification program	1 (4%)	0 (0%)	1 (2.2%)
Associate's degree	0 (0%)	2 (9%)	2 (4.3%)
Bachelor's degree	9 (36%)	8 (36%)	17 (39.5%)
Master's degree	10 (40%)	9 (40%)	19 (41.3%)
Medical/Doctoral degree	4 (28%)	3 (13%)	7 (15.2%)
Discipline			
Psychology	4 (16%)	5 (21%)	9 (19.6%)
Developmental-Behavioral Pediatrics	3 (12%)	2 (9%)	5 (10.9%)
Speech-Language Pathology	2 (8%)	2 (9%)	4 (8.7%)
Genetic Counseling	2 (8%)	2 (9%)	4 (8.7%)
Family advocacy	2 (8%)	3 (13%)	5 (10.9%)
Adult Services	1 (4%)	0 (0%)	1 (2.2%)
Advocacy	1 (4%)	1 (4%)	2 (4.3%)
Applied Behavior Analysis	1 (4%)	0 (0%)	1 (2.2%)
Audiology	1 (4%)	1 (4%)	2 (4.3%)
Disability Studies	1 (4%)	0 (0%)	1 (2.2%)
Education	1 (4%)	0 (0%)	1 (2.2%)
Family Medicine	1 (4%)	0 (0%)	1 (2.2%)
Occupational Therapy	1 (4%)	2 (9%)	1 (2.2%)
Physical therapy	1 (4%)	1 (9%)	2 (4.3%)
Public Health	1 (4%)	1 (4%)	2 (4.3%)
Social Work	1 (4%)	2 (9%)	3 (6.5%)

Mean scores on the Perceived Knowledge and Application of DEIJ (Table 4) increased across the LEND training year [*t*(45) = 4.94, *p* < .001, M_diff_ = .45, *Cohen's D* = 0.73]. While trainees’ Perceived Knowledge increased [*t*(45) = 5.84, *p* < .001, M_diff_ = .59, *Cohen's D* = 0.86], trainees’ comfort with addressing prejudicial statements and combating “-isms” (Application) did not increase over the LEND year [*t*(45) = 1.74, *p* = .09, *M_diff_* = 0.17, Cohen's D = 0.26].

**Table 4:** Pre- and post-scores on the perceived knowledge and application of DEIJ survey.

Perceived knowledge domain prompt	Pre, mean (SD)	Post, mean (SD)
“I am aware of biases that I have towards individuals with backgrounds different from my own”	4.91 (1.02)	5.33 (.60)
“I can identify -isms (such as racism, classism, Eurocentrism) that are used in policies and by institutions (e.g., government).”	4.63 (.95)	5.30 (.70)
“I am aware of my identities that give me privilege.”	5.07 (.98)	5.30 (.89)
“I understand the ways in which my cultural background influences my views of the world.”	5.07 (.84)	5.43 (.54)
“I understand intersectionality within the disability community.”	4.46 (1.57)	5.24 (.67)
“I understand the impacts of ableism on individuals with disabilities’ everyday lives.”	4.43 (1.39)	5.46 (.59)
Perceived Knowledge Domain Average	4.76 (.71)**	5.35 (.45)**
Application Domain Prompt	Pre, Mean (SD)	Post, Mean (SD)
“I feel comfortable confronting prejudicial statements and microaggressions in my everyday life.”	4.52 (1.13)	4.89 (.90)
“I am committed to fighting against -isms (such as ethnocentrism, heterosexism, genderism) in my everyday life.”	5.59 (.65)	5.54 (.62)
“I feel comfortable discussing -isms (such as ageism, sexism, ableism) in my everyday life (e.g., personal, professional, with family, with friends).	5.02 (1.11)	5.24 (.77)
Application Domain Average	5.05 (.77)	5.22 (.62)
Perceived Knowledge and Application Average	4.86 (.64)**	5.31 (.45)**

** *p* < .001.

**Figure 1:**
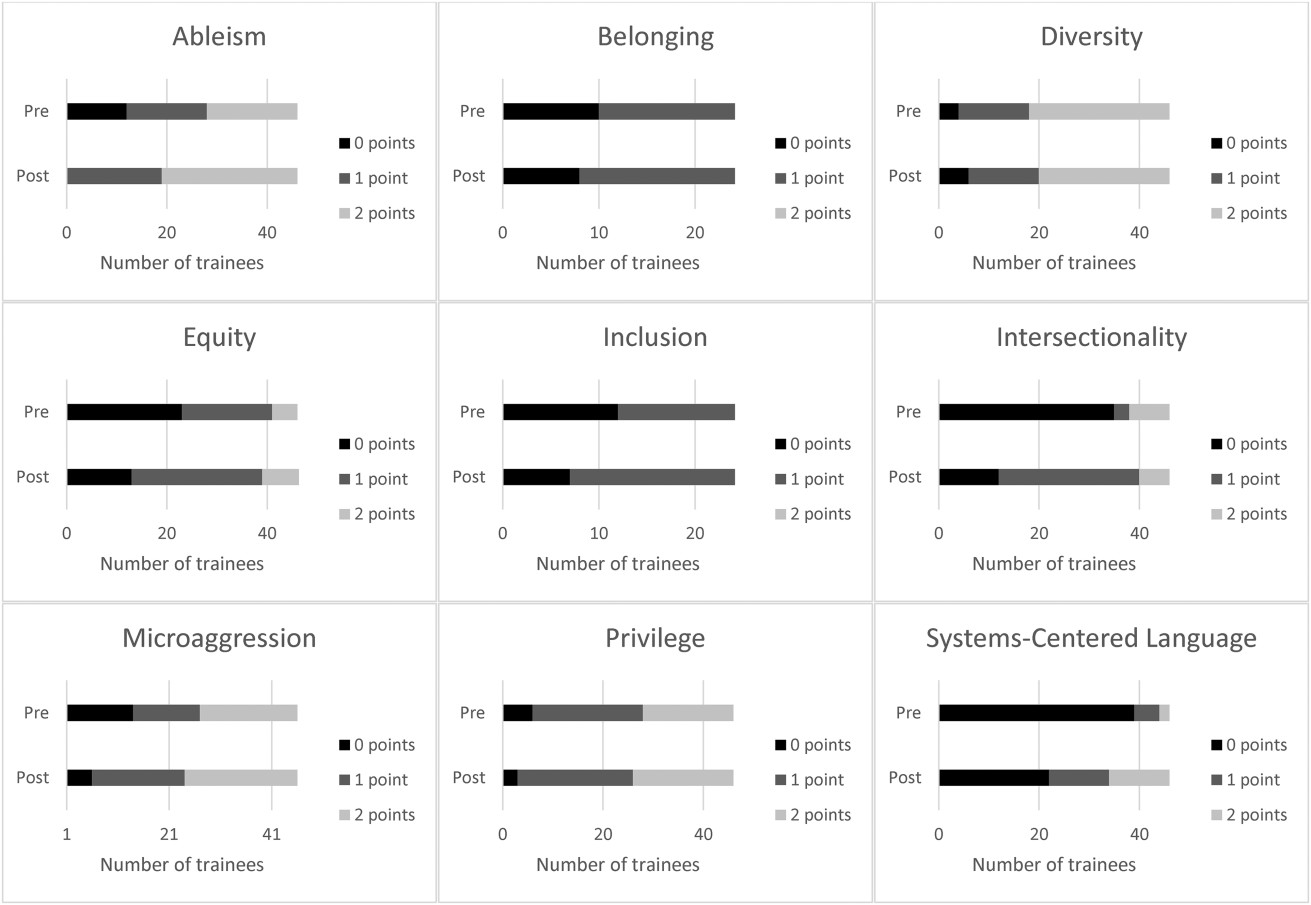
Number of 0-point, 1-point, and 2-point responses to trainees' Diversity Equity Inclusion and Justice term definitions before and after Leadership Education in Neurodevelopmental and Related Disabilities.

Trainees’ summed scores following participation in LEND increased [*t*(45) = 4.71, *p* < .001, *M_diff_* = 2.37, *Cohen's D* = 0.70; See Figure 1]. Before LEND, 37% of trainees’ definitions were given 0 points in comparison to 19% after LEND. The percentage of trainees’ responses labeled “partially correct” increased from 34% before LEND to 45% after LEND. The percentage of trainees’ responses labeled “correct” increased from 28% to 36%.

When examining responses to individual terms, most trainees (*n* = 26, 57%) described what “equity” is (i.e., fair distribution of resources based on individual need) after LEND participation; however, only seven (15%) trainees gave a 2-point response, acknowledging why equity is necessary (i.e., equity is needed to prevent structural inequality leading to oppression). Similarly, half of trainees described “privilege” as an advantage (*n* = 23, 50%), whereas 20 (44%) trainees gave a 2-point response, describing that privilege is an advantage that is unearned or innate. With “intersectionality,” 28 (61%) trainees reported that intersectionality is an interaction or intersection of identity groups; however, only six (13%) trainees acknowledged that the intersection of identities leads to privilege and/or marginalization (2-point response). Most trainees (*n* = 27, 59%) provided a 2-point response for “ableism” though the remaining 19 (41%) trainees did not provide thorough descriptions of ableism. For example, one trainee described that ableism is discrimination against individuals with intellectual disability, thereby incorrectly excluding other types of disabilities in their response. Twenty-two (48%) trainees provided 2-point responses when asked to define “microaggressions.” The remaining 24 trainees (52%) provided incorrect definitions (e.g., “passive aggressive”) or partially correct responses (e.g., did not acknowledge that microaggressions cause harm, did not differentiate microaggression from macroaggression). Many trainees (*n* = 22, 48%) were unable to define “systems-centered language” after LEND. Twelve (26%) understood that systems-centered language involves language that describes systems rather than individuals, but only 12 (26%) trainees described that the term holds systems accountable for perpetrating oppression rather than blaming individuals for existing disparities (2-point response). With “diversity,” 14 (30%) trainees did not provide thorough definitions of the term, such as referring to but not defining “differences” (e.g., answer limited to “people being different from one another and celebrating our differences”). Nearly half of the trainees described “inclusion” as including people of different backgrounds and/or ability statuses (*n* = 22, 48%); seventeen (37%) trainees described that it is important to ensure the environment or space allows for participation or inclusion (2-point response). Finally, regarding “belonging,” 26 (57%) trainees expressed the inward feeling of being a part of a group or being able to be one's authentic self. Similar to “inclusion,” only 12 (26%) trainees gave a 2-point response, describing the importance of the environment or community's contribution to that sense of safety.

## Discussion

We found that trainees’ perceived knowledge about DEIJ increased after participation in LEND, with a large effect size. Before LEND participation, trainees were likely to “Somewhat Agree” they understood important DEIJ concepts, and after LEND, trainees were likely to “Agree” they understood important DEIJ concepts. Similar to previous research, we did not identify an increase in trainees’ comfort with application of DEIJ [i.e., addressing systemic inequity and prejudice in their everyday lives (13)]. Trainees responded on average between a 5 (“Agree”) and 6 (“Completely Agree”) on a 6-point Likert scale to two of the three application-based questions, possibly reflecting social desirability bias, which is common in scales assessing cultural humility (14). The high baseline scores may contribute to a ceiling effect, limiting the potential for measurable improvement. Additionally, the application questions asked trainees about their comfort in addressing prejudicial statements and “-isms” in their everyday life, which can be influenced by many factors, including power differentials, fear of negative consequences and retaliation, and being in an unsafe culture or environment. It may be helpful to explore when and how trainees confront microaggressions, prejudicial statements, and inequity through qualitative interviews or observation. Though the LEND program uses case-based learning, small group discussions, and role play to promote behavior change, trainees need additional practice and experience to increase their comfort with applying DEIJ concepts in their everyday life.

When defining DEIJ terms, trainees’ overall knowledge increased, with a medium effect size. Most trainees gained a basic understanding of important DEIJ terms, but only about one-third of responses were in-depth, complete definitions following LEND program participation. Trainees appeared to be most knowledgeable about ableism, diversity, and microaggressions after LEND completion, with 50% or more trainees providing 2-point definitions for these responses. By contrast, less than one-third of trainees provided 2-point responses for equity, intersectionality, systems-centered language, inclusion, and belonging. For these terms, trainees often failed to articulate systemic implications. For example, many trainees did not describe that equity is needed to mitigate the impacts of systemic oppression, that the intersection of identity leads to privilege or marginalization, or that the environment and culture need to shift to facilitate inclusion and belonging. These findings highlight that the LEND program needs to focus more on structural competency to facilitate trainees’ understanding of systemic factors that contribute to oppression.

This study should be considered in the context of its limitations. First, the cohort's demographic characteristics (i.e., the LEND trainees) may have influenced our findings. Most trainees were White women with some graduate training, pursuing service careers dedicated to working with individuals with disabilities. Therefore, most trainees have multiple privileged identities, and their career interests may increase their motivation to learn about DEIJ, especially as it relates to better supporting diverse individuals with disabilities.

Second, there are few validated assessment measures to understand individuals’ knowledge and application of DEIJ. As such, we developed measures for use in this program. Future research should assess the psychometric properties of our measures, with a larger sample size. We addressed some limitations of previous self-report based studies by rating changes in trainees’ understanding of important DEIJ concepts. However, we also used self-reported measures of changes in knowledge and skills. It is possible trainees responded more positively about their knowledge, especially during the post-assessment, because they were aware that the training program was designed to enhance these competencies over the year (49). Additionally, though trainees’ understanding of DEIJ terms increased throughout the training year, trainees continued to demonstrate gaps in their knowledge of how structural inequality is related to systems-centered language, intersectionality, equity, inclusion, and belonging. Trainees’ gaps in knowledge will be used to continually modify the LEND training curriculum to improve trainees’ understanding of DEIJ.

A major limitation of the study we hope to address in future studies is identifying whether changes in knowledge of DEIJ concepts, self-reported knowledge, and self-reported skills translate to changes in behaviors, leadership or patient care. As we adapt the curriculum, we can assess increases in trainees’ ability to provide culturally responsive care and skills in addressing microaggressions and structural inequality using observational methods (e.g., observations of patient encounters, team meetings) and patient-reported measures. Finally, our study examined only short-term gains in knowledge and application, and we plan to assess if improvements are maintained over time.

## Conclusion

The LEND curriculum focuses on developing MCH competencies, which are designed to progress from self-awareness and reflection to leadership skills that can be applied to trainees’ communities and at the systems-level. Our study demonstrated that the LEND training program increased trainees’ perceived knowledge of DEIJ and ability to define important DEIJ terms and concepts. We aim to continuously improve the LEND curriculum to support trainees’ skills in addressing structural inequality. LEND trainees are tomorrow's leaders who will be responsible for the design and implementation of interventions, programs, and research that address health equity. Although achieving health equity requires systems level change, these changes cannot be accomplished without starting at the individual, trainee level. Individuals with disabilities and their families are marginalized and face discrimination in healthcare. Essential to driving progress in this area is effective measurement of workforce development and training outcomes. Our study is the beginning of systematic efforts to improve trainees’ cultural responsiveness. Future iterations of this study will examine the validity of our measures, use objective outcomes to examine changes in trainees’ behaviors, and collaborate with sites across the LEND Network.

## Data Availability

The raw data supporting the conclusions of this article will be made available by the authors, without undue reservation.

## References

[1] Alberto CK Kemmick Pintor J McKenna RM Roby DH Ortega AN. Racial and ethnic disparities in provider-related barriers to health care for children in California after the ACA. Glob Pediatr Health. (2019) 6:2333794×19828356. 10.1177/2333794X19828356 PMC6376499 30793014

[2] Soylu TG Elashkar E Aloudah F Ahmed M Kitsantas P. Racial/ethnic differences in health insurance adequacy and consistency among children: evidence from the 2011/12 national survey of Children's Health. J Public Health Res. (2018) 7(1):1280. 10.4081/jphr.2018.1280 29780766 PMC5941257

[3] Fiscella K Sanders MR. Racial and ethnic disparities in the quality of health care. Annu Rev Public Health. (2016) 37:375–94. 10.1146/annurev-publhealth-032315-021439 26789384

[4] Maina IW Belton TD Ginzberg S Singh A Johnson TJ. A decade of studying implicit racial/ethnic bias in healthcare providers using the implicit association test. Soc Sci Med. (2018) 199:219–29. 10.1016/j.socscimed.2017.05.009 28532892

[5] Williams DR Lawrence JA Davis BA. Racism and health: evidence and needed research. Annu Rev Public Health. (2019) 40:105–25. 10.1146/annurev-publhealth-040218-043750 30601726 PMC6532402

[6] Krahn GL Walker DK Correa-De-Araujo R. Persons with disabilities as an unrecognized health disparity population. Am J Public Health. (2015) 105 Suppl 2(Suppl 2):S198–206. 10.2105/AJPH.2014.302182 25689212 PMC4355692

[7] Mink MD Lindley LL Weinstein AA. Stress, stigma, and sexual minority status: the intersectional ecology model of LGBTQ health. J Gay Lesbian Soc Serv. (2014) 26(4):502–21. 10.1080/10538720.2014.953660

[8] Sentell T Braun KL. Low health literacy, limited English proficiency, and health status in Asians, Latinos, and other racial/ethnic groups in California. J Health Commun. (2012) 17:82–99. 10.1080/10810730.2012.712621 23030563 PMC3552496

[9] Chang CD. Social determinants of health and health disparities among immigrants and their children. Curr Probl Pediatr Adolesc Health Care. (2019) 49(1):23–30. 10.1016/j.cppeds.2018.11.009 30595524

[10] Govere L Govere EM. How effective is cultural competence training of healthcare providers on improving patient satisfaction of minority groups? A systematic review of literature. Worldviews Evid Based Nurs. (2016) 13(6):402–10. 10.1111/wvn.12176 27779817

[11] Garcia KA Wippold GM Goodrum NM Williams MM Kloos B. Bridging health self-efficacy and patient engagement with patient-centered culturally sensitive health care for black American adults. J Community Psychol. (2024). 10.1002/jcop.23147 39213672

[12] Patneaude A Kett J. Cultural responsiveness and palliative care during the COVID-19 pandemic. Palliat Med Rep. (2020) 1(1):171–3. 10.1089/pmr.2020.0049 34223473 PMC8241343

[13] Brottman MR Char DM Hattori RA Heeb R Taff SD. Toward cultural competency in health care: a scoping review of the diversity and inclusion education literature. Acad Med. (2020) 95(5). Available online at: https://journals.lww.com/academicmedicine/Fulltext/2020/05000/Toward_Cultural_Competency_in_Health_Care__A.37.aspx 10.1097/ACM.0000000000002995 31567169

[14] Larson KE Bradshaw CP. Cultural competence and social desirability among practitioners: a systematic review of the literature. Child Youth Serv Rev. (2017) 76:100–11. 10.1016/j.childyouth.2017.02.034

[15] Boulet SL Boyle CA Schieve LA. Health care use and health and functional impact of developmental disabilities among US children, 1997–2005. Arch Pediatr Adolesc Med. (2009) 163(1):19–26. 10.1001/archpediatrics.2008.506 19124699

[16] Arim RG Miller AR Guèvremont A Lach LM Brehaut JC Kohen DE. Children with neurodevelopmental disorders and disabilities: a population-based study of healthcare service utilization using administrative data. Dev Med Child Neurol. (2017) 59(12):1284–90. 10.1111/dmcn.13557 28905997

[17] Lee D Pollack SW Mroz T Frogner BK Skillman SM. Disability competency training in medical education. Med Educ Online. (2023) 28(1):2207773. 10.1080/10872981.2023.2207773 37148284 PMC10167870

[18] Smeltz L Havercamp SM Meeks L. Aspiring to disability consciousness in health professions training. AMA J Ethics. (2024) 26(1):E54–61. 10.1001/amajethics.2024.54 38180859

[19] Sharby N Martire K Iversen MD. Decreasing health disparities for people with disabilities through improved communication strategies and awareness. Int J Environ Res Public Health. (2015) 12(3):3301–16. 10.3390/ijerph120303301 25809511 PMC4377965

[20] Ali A Scior K Ratti V Strydom A King M Hassiotis A. Discrimination and other barriers to accessing health care: perspectives of patients with mild and moderate intellectual disability and their carers. PLoS One. (2013) 8(8):e70855. 10.1371/journal.pone.0070855 23951026 PMC3741324

[21] Temple JB Kelaher M Brooke L Utomo A Williams R. Discrimination and disability: types of discrimination and association with trust, self-efficacy and life satisfaction among older Australians. Australas J Ageing. (2020) 39(2):122–30. 10.1111/ajag.12747 31749271

[22] VanPuymbrouck L Friedman C Feldner H. Explicit and implicit disability attitudes of healthcare providers. Rehabil Psychol. (2020) 65(2):101–12. 10.1037/rep0000317 32105109 PMC9534792

[23] Drainoni M-L Lee-Hood E Tobias C Bachman SS Andrew J Maisels L. Cross-disability experiences of barriers to health-care access: consumer perspectives. J Disabil Policy Stud. (2006) 17(2):101–15. 10.1177/10442073060170020101

[24] Neri MT Kroll T. Understanding the consequences of access barriers to health care: experiences of adults with disabilities. Disabil Rehabil. (2003) 25(2):85–96. 10.1080/0963828021000007941 12554383

[25] Crenshaw K. Demarginalizing the intersection of race and sex: a black feminist critique of antidiscrimination doctrine, feminist theory and antiracist politics. University of Chicago Legal Forum. (1989) 1989(1):8. https://chicagounbound.uchicago.edu/uclf/vol1989/iss1/8

[26] Dorsey Holliman B Stransky M Dieujuste N Morris M. Disability doesn't Discriminate: health inequities at the intersection of race and disability. Frontiers in Rehabilitation Sciences. (2023) 4:1075775. 10.3389/fresc.2023.1075775 37484601 PMC10357509

[27] Fredriksen-Goldsen KI Kim H-J Barkan SE. Disability among lesbian, gay, and bisexual adults: disparities in prevalence and risk. Am J Public Health. (2012) 102(1):e16–21. 10.2105/AJPH.2011.300379 22095356 PMC3490559

[28] Warner DF Brown TH. Understanding how race/ethnicity and gender define age-trajectories of disability: an intersectionality approach. Soc Sci Med. (2011) 72(8):1236–48. 10.1016/j.socscimed.2011.02.034 21470737 PMC3087305

[29] Wolbring G Deloria R. Health equity and health inequity of disabled people: a scoping review. Sustainability. (2024) 16(16). 10.3390/su16167143

[30] HRSA. MCH Leadership competencies. (2018). Available online at: https://mchb.hrsa.gov/programs-impact/focus-areas/building-mch-leaders-mch-workforce/leadership-competencies (accessed November 27, 2021).

[31] Humphreys BP Kurtz AJ Portrie C Couse LJ Hajnaghizadeh F. Advancing leadership skills: a multiyear examination of LEND trainee self-efficacy growth. Matern Child Health J. (2018) 22:1377–83. 10.1007/s10995-018-2582-2 30006731

[32] Smith JD Nidey N Chödrön GS Czyzia J Donahue ML Ford K A quality improvement network for interdisciplinary training in developmental disabilities. Pediatrics. (2022) 150(6):e2022058236. 10.1542/peds.2022-058236 36349516

[33] Weber S Williams-Arya P Bowers K Wamsley F Doarn CR Smith J. Effectiveness of interdisciplinary leadership training for early career professionals in the field of developmental disabilities. Matern Child Health J. (2021) 25(7):1036–42. 10.1007/s10995-021-03166-8 33961209

[34] Bishop L Harris AB Rabidoux PC Laughlin SF McLean KJ Noll RB. A model to evaluate interprofessional training effectiveness: feasibility and five-year outcomes of a multi-site prospective cohort study. Matern Child Health J. (2022) 26(8):1622–31. 10.1007/s10995-022-03421-6 35583590 PMC9513993

[35] Tervalon M Murray-García J. Cultural humility versus cultural competence: a critical distinction in defining physician training outcomes in multicultural education. J Health Care Poor Underserved. (1998) 9(2):117–25. 10.1353/hpu.2010.0233 10073197

[36] Metzl JM Petty J Olowojoba OV. Using a structural competency framework to teach structural racism in pre-health education. Soc Sci Med. (2018) 199:189–201. 10.1016/j.socscimed.2017.06.029 28689630

[37] Agness-Whittaker CF Macedo L. Aging, culture, and health communication: exploring personal cultural health beliefs and strategies to facilitate cross-cultural communication with older adults. MedEdPORTAL. (2016) 12:10374. 10.15766/mep_2374-8265.10374

[38] Mihalic AP Morrow JB Long RB Dobbie AE. A validated cultural competence curriculum for US pediatric clerkships. Patient Educ Couns. (2010) 79(1):77–82. 10.1016/j.pec.2009.07.029 19699600

[39] Vyas D Caligiuri FJ. Reinforcing cultural competency concepts during introductory pharmacy practice experiences. Am J Pharm Educ. (2010) 74(7). 10.5688/aj7407129 PMC2972524 21088735

[40] Lim RF Wegelin J Hua LL Kramer EJ Servis ME. Evaluating a lecture on cultural competence in the medical school preclinical curriculum. Acad Psychiatry. (2008) 32:327–31. 10.1176/appi.ap.32.4.327 18695035

[41] Poirier TI Butler LM Devraj R Gupchup GV Santanello C Christopher Lynch J. A cultural competency course for pharmacy students. Am J Pharm Educ. (2009) 73(5):81. 10.5688/aj730581 19777096 PMC2739064

[42] Ring JM. Psychology and medical education: collaborations for culturally responsive care. J Clin Psychol Med Settings. (2009) 16:120–6. 10.1007/s10880-008-9141-8 19153824

[43] Pilcher ES Charles LT Lancaster CJ. Development and assessment of a cultural competency curriculum. J Dent Educ. (2008) 72(9):1020–8. 10.1002/j.0022-0337.2008.72.9.tb04576.x 18768444

[44] Lim RF Diamond RJ Chang JB Primm AB Lu FG. Using non-feature films to teach diversity, cultural competence, and the DSM-IV-TR outline for cultural formulation. Acad Psychiatry. (2008) 32:291–8. 10.1176/appi.ap.32.4.291 18695030

[45] Westberg SM Bumgardner MA Lind PR. Enhancing cultural competency in a college of pharmacy curriculum. Am J Pharm Educ. (2005) 69(5). 10.5688/aj690582

[46] Kruse JA Collins JL Vugrin M. Educational strategies used to improve the knowledge, skills, and attitudes of health care students and providers regarding implicit bias: an integrative review of the literature. Int J Nurs Stud Adv. (2022) 4:100073. 10.1016/j.ijnsa.2022.100073 38745633 PMC11080399

[47] DEIJ Glossary. (2022). Available online at: https://www.hartford.edu/about/diversity-inclusion/deij-learning-resources/deij-glossary.aspx#accordion-group-1-section-24-label (accessed March 27, 2023).

[48] Baron IS. Test review: wechsler intelligence scale for children-fourth edition (WISC-IV). Child Neuropsychol. (2005) 11(5):471–5. 10.1080/09297040590951587 16306021

[49] Althubaiti A. Information bias in health research: definition, pitfalls, and adjustment methods. J Multidiscip Healthc. (2016) 9:211–7. 10.2147/JMDH.S104807 27217764 PMC4862344

